# Association of Common Mental Disorders and Quality of Life with the Frequency of Attendance in Slovenian Family Medicine Practices: Longitudinal Study

**DOI:** 10.1371/journal.pone.0054241

**Published:** 2013-01-16

**Authors:** Janez Rifel, Igor Švab, Polona Selič, Danica Rotar Pavlič, Irwin Nazareth, Josip Car

**Affiliations:** 1 Department of Family Medicine, Medical Faculty, University of Ljubljana, Ljubljana, Slovenia; 2 Department of Primary Care and Population Health, University College London, London, United Kingdom; 3 Global eHealth Unit, Department of Primary Care and Public Health, School of Public Health, Imperial College London, London, United Kingdom; University of Pennsylvania, United States of America

## Abstract

**Background:**

Most research on frequent attendance has been cross-sectional and restricted to one year attendance rates. A few longitudinal studies suggest that frequent attendance is self-limiting. Frequent attenders are more likely to have social and psychiatric problems, medically unexplained physical symptoms, chronic somatic diseases (especially diabetes) and are prescribed more psychotropic medication and analgesics.

**Research Question:**

To describe the attendance rates in a longitudinal study and to test if depression, panic syndrome, other anxiety syndrome, alcohol misuse and general quality of life are associated with frequent attendance in next two consecutive years.

**Methods:**

1118 consecutive family practice attendees, aged 18 to 75 years from randomly selected family medicine practices were recruited at baseline and followed up at 12 and 24 months. We identified frequent attenders in the top 10 centile within one year. Using a multivariate model, we ascertained if presence of common mental disorders and quality of life assessed at baseline in 2003 predict frequent attendance in 2004 and 2005.

**Results:**

40% of frequent attenders continue to be frequent attenders in the following year and 20% of the frequent attenders were so for the 24 month period. Lower physical scores on the SF-12 questionnaire were strongly associated with future frequent attendance at 12 and 24 months. There was a trend for people with greater than elementary school education to be less likely to become frequent attenders at both 12 and 24 months. For other variables these effects were less consistent. Presence of major depression, panic syndrome, other anxiety syndrome and alcohol misuse were not predictive of frequent attendance in the following two years.

**Conclusion:**

Low physical quality of life is strongly predictive of higher frequent attendance and similar finding was observed for people with lower educational level but further confirmatory research is required to establish this association.

## Introduction

A study from the UK estimated that family physicians devote 80% of their time to 20% of their patients and one in six consultations occur with the top 3% of attenders [1]. Although there is no agreed definition of frequent attendance in family medicine [2], several studies define frequent attendance as the top 10 per centiles of attendance rates over one year adjusted for age and sex [3]. This makes it possible to make meaningful comparisons between different periods, practices, regions and countries [2], [3]. Most research on frequent attendance has been cross-sectional and restricted to one year attendance rates. There are very few longitudinal studies and these suggest that frequent attendance is self-limiting [3] and only 20 to 30% of frequent attenders attend frequently in the following year [4], [5].

Persistent frequent attendance (i.e. people who attend frequently for periods greater than one year) [3] is observed in the elderly and those with chronic illnesses whereas short-term frequent attendance is more common in people with self-limiting conditions such as mild depression and pregnancy [5]. Persistent frequent attenders are more likely to have social and psychiatric problems, medically unexplained physical symptoms, chronic somatic diseases (especially diabetes) and are prescribed more psychotropic medication and analgesics [3], [6].

Most of the literature on frequent attendance has come from countries where practitioners manage an electronic list of patients such as the UK, Scandinavian countries and countries with health maintenance organisations such as the USA [2], [7]. Previous work in Slovenia on frequent attendance in general practice [8], [9] includes a cross-sectional study in which patients in the top quartile of yearly contact rates were associated with lower education levels, chronic health problems and higher use of other health services.

In our study we aimed to describe the attendance rates in a longitudinal study and test if depression, panic disorders, other anxiety disorders, alcohol misuse and general quality of life predict frequent attendance in the next two consecutive years.

## Methods

### Ethical Approval

The study was approved by the National Medical Ethics Committee of the Republic of Slovenia. Participants provided their written informed consent to participate in the study.

### Setting and Participants

We randomly selected 60 family physicians from the Slovenian national register of family medicine physicians. Each family physician recruited 10 to 20 consecutive family practice attendees aged 18 to 75 years. Our plan as a part of the PREDICT D study was to recruit 1100 participants [10]. Participants were recruited at the end of year 2003 and followed up 12 months later in 2004 and 24 months later in 2005. The participating family medicine practices were selected from urban and rural settings and served a population with diverse socio-economic and ethnic characteristics.

1396 primary care attendees were approached in 2003 and 275 of them refused to participate in the study.

In the year 2008 we obtained consultation rate data for 816 patients (out of 1018 recruited) in 2003, consultation rate data for 760 patients in 2004 and consultation rate data for 527 patients in 2005 that were not lost to follow up.

### Instruments Used in the Study

Baseline interviews were carried out by 36 trained interviewers. We assessed the presence of depression (by Composite International Diagnostic Interview) [10], [11], [12], the presence of panic syndrome and other anxiety syndrome (by Patient Health Questionnaire) [13], quality of life (measured with physical and mental score of SF-12) [14], and alcohol misuse (AUDIT score) [15]. The instruments have established validity and reliability [10].

Information on age, gender, educational level, occupational status and whether the patient is living alone or not was also gathered. In 2008, the participating practitioner clinics were contacted and we obtained their data on the consultation rates of participating patients in years 2003, 2004 and 2005.

### Statistical Analysis

We defined frequent attenders among participating patients as the top 10 centiles within a time frame of one year. We followed up a cohort of participants for two years to ascertain how many patients continued to be frequent attenders for more than one year. Using a multivariate model we identified those factors assessed at baseline in 2003 that predicted frequent attendance in 2004 and 2005.

## Results

The main reasons for refusal were lack of participants’ interest (N = 76), the researcher inability to contact them (N = 41) and inadequate time (N = 27). Three attendees were not eligible, because they were older than 75 years. In total 1118 attendees participated in 2003. In the year 2004 1036 participants were re-interviewed which is 93% of the baseline number and in 2005, 784 (70%) participants were re-interviewed.

### Demographic Data of the Non-respondents

Even if a person refused to participate in the study we requested information on their age and gender. Table 1 present age and rate of women in the sample of participating attendees and attendees who refused to participate.

**Table 1:** Age and percent of women in the groups of respondents and non-respondents.

	Age (mean, [SD])	Gender (percent of women)
Participating attendees (n = 1118)	48,7 [14], [4]	63,4
Refused to participate (n = 270)	50,0 [14,0]	59,6

There was trend for attendees who refused to participate in the study to be older and to be men but there were no statistically significant differences between the groups of participating and non-participating attendees.

Losses to follow-up were mainly due to physician’s unwillingness to continue to support the study due to time pressures. In the years 2003, 2004 and 2005, frequent attenders based on the 10^th^ centile cut off were participants who in the preceding year visited their physician 18 times or more/year in 2003, 17 or more/year in 2004 and 15 or more/year in 2005 (see Table 2). Using this definition there were 105 frequent attendees in 2003, 94 in 2004 and 63 in 2005.

**Table 2:** Number and percentage of frequent attenders in years 2003–2005.

	Number of frequent attenders	Percentage of frequent attenders	10^th^ centile cut off(number of visits per year)
2003 (n = 816)	105	12,4	18
2004 (n = 760)	94	11,9	17
2005 (n = 527)	63	11,6	15

**Figure 1:**
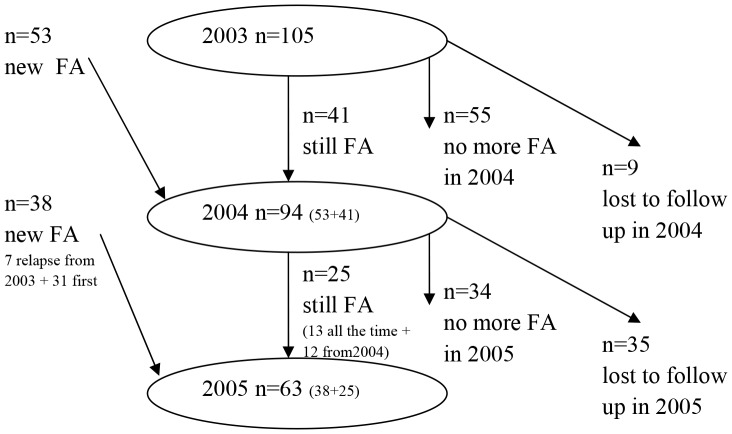
Flow diagram of frequent attenders (FA) from 2003 to 2005.

In Figure 1 we present the numbers of frequent attenders from 2003 to 2005. Only 13 participants were frequent attenders over the three year period. In the year 2004, 9 frequent attenders from 2003 were lost to follow up. From the remaining 96 frequent attenders in 2003, 41 participants remained frequent attenders in 2004. In 2005, 35 frequent attenders from 2004 were lost to follow up. Of the remaining 59 frequent attenders from 2004, 25 participants remained frequent attenders in the year 2005. In each successive year around 42% of frequent attenders continued to attend frequently (41 from 96 patients and 25 from 59 patients for the years 2004 and 2005 respectively).

In Table 3 we present gender, educational level and age at baseline. In 2003, frequent attenders were more often older, men and people of lower educational level.

**Table 3:** Demographic data for the year 2003 (n = 847).

	non-frequent		frequent attenders		total	
	n = 742	%	n = 105	%	n = 847	%
Gender						
female	470	63,3	62	59,0	532	62,8
male	272	36,7	43	41,0	315	37,2
Education						
elementary school or less	155	20,9	33	31,4	188	22,2
vocational	163	22,0	34	32,4	197	23,3
secondary school	291	39,2	28	26,7	319	37,7
higher school or university	133	17,9	10	9,5	143	16,9
Age in years (Mean ± SD)	48,5±14,5		50,6±12,5		48,7±14,3	

In Tables 4 and 5 we present demographic data for the sample followed up in the years 2004 and 2005.

**Table 4:** Demographic data for the year 2004 (n = 791).

	non-frequent		frequent attenders		total	
	n = 697	%	n = 94	%	n = 791	%
Gender						
female	439	63,0	53	56,4	492	62,2
male	258	37,0	41	43,6	299	37,8
Education						
elementary school or less	132	18,9	31	33,0	163	20,6
Vocational	165	23,7	26	27,7	191	24,1
secondary school	270	38,7	27	28,7	297	37,5
higher school or university	130	18,7	10	10,6	140	17,7
Age in years (Mean ± SD)	48,5±14,5		48,1±12,3		48,5±14,3	

**Table 5:** Demographic data for the year 2005 (n = 542).

	non-frequent		frequent attenders		total	
	n = 479	%	n = 63	%	n = 542	%
Gender						
female	296	61,8	39	61,9	335	61,8
male	183	38,2	24	38,1	207	38,2
Education						
elementary school or less	81	16,9	23	36,5	104	19,2
vocational	102	21,3	11	17,5	113	20,8
secondary school	197	41,1	23	36,5	220	40,6
higher school or university	99	20,7	6	9,5	105	19,4
Age in years (Mean ± SD)	49,8±14,5		55,3±13,5		50,4±14,5	

In years 2004 and 2005 the educational level of frequent attenders was also lower than total sample. In 2004 frequent attenders are younger. In 2005, percentage of men in the group of frequent attenders is the same as in the total sample.

In Table 6, we present the results from the multivariate model for the prediction of frequent attendance. Lower physical score (SF-12) is a powerful predictor of future frequent attendance at 12 and 24 months. A lower mental health score (SF-12) predicts frequent attendance after 24 months but not after 12 months.

**Table 6:** Multivariate model showing effects of risk factors for frequent attendance in the two following years after assessment of risk factors (df = degrees of freedom, OR = odds ratio, CI = confidence intervals).

	Frequent attenders 2004 (Model χ^2^ = 102.127, df = 14, p<0.001)			Frequent attenders 2005 (Model χ^2^ = 65.279, df = 14, p<0.001)		
	?^2^	OR (95%CI)	p	?^2^	OR (95%CI)	p
major depression	3.47	2.81 (0.95–8.35)	0.062	0.33	0.63 (0.13–3.02)	0.564
panic syndrome	0.38	0.73 (0.27–1.98)	0.539	1.19	1.91 (0.60–6.10)	0.276
other anxiety syndrome	1.57	2.04 (0.67–6.21)	0.211	0.11	1.28 (0.30–5.40)	0.741
audit score	2.24	0.92 (0.83–1.03)	0.135	3.47	1.14 (0.99–1.30)	0.063
SF-12mentalscore	1.24	0.99 (0.96–1.01)	0.266	6.17	0.96 (0.93–0.99)	0.013
SF-12physical score	42.83	0.93 (0.91–0.95)	<0.001	25.83	0.93 (0.91–0.96)	<0.001
age	2.68	0.98 (0.96–1.00)	0.101	1.01	1.01 (0.99–1.04)	0.314
male gender	4.59	1.80 (1.05–3.10)	0.032	0.15	1.14 (0.58–2.24)	0.699
Education						
elementary school or less		1.00			1.00	
vocational	3.67	0.52 (0.27–1.02)	0.056	4.36	0.40 (0.17–0.95)	0.037
secondary school	4.15	0.51 (0.27–0.97)	0.042	3.22	0.51 (0.25–1.06)	0.073
higher school or university	2.83	0.49 (0.21–1.12)	0.093	6.42	0.26 (0.09–0.74)	0.011
living alone	0.51	0.72 (0.29–1.77)	0.473	2.63	2.05 (0.86–4.88)	0.105
Occupation						
employed		1.00			1.00	
housewife, student, unemployed	5.44	0.23 (0.07–0.79)	0.020	0.62	0.58 (0.15–2.25)	0.431
pensioner, unable to work	2.10	0.60 (0.31–1.19)	0.147	1.26	0.65 (0.31–1.38)	0.262

Nagelkerke R^2^ = 0.234 Nagelkerke R^2^ = 0.221.

Male gender was significantly associated with frequent attendance after 12 months but not 24 months. At 12 months there is a reduced likelihood of frequent attendance (OR = 0.51, 95 CI, 0.27, 0.97) for people with secondary school and a trend of this effect for people with vocational training. At 24 months the effect for vocational training becomes significant 0.40 (0.17–0.95) and this hold true for those with university education 0.26 (0.09–0.74) but the effect for those with secondary education is lost. Being a housewife, a student or unemployed is a protective factor for frequent attendance at 12 months (0.23 95% CI 0.07–0.79) but not at 24 months.

Presence of depression, panic syndrome, other anxiety syndrome, AUDIT score, age and living alone had no significant effects on frequent attendance after 12 and after 24 months.

## Discussion

This is the first longitudinal study of frequent attendance in Slovenian primary care showing that more than 40% of frequent attenders remain frequent attenders in the following year, which is higher than rate reported in the literature (20–30%). Around 20% of frequent attenders in the year 2005, the last year of our follow up, were also frequent attenders in previous two years. Low physical score (suggestive of poor physical health) were strongly predictive of frequent attendance at 12 and 24 months. There was no consistent predictive trend with respect to any of the other variables. The presence of major depression, panic syndrome, other anxiety syndrome and alcohol misuse did not predict frequent attendance in the following two years.

The two dimensions of quality of life measured by the SF-12 questionnaire the physical and mental score, showed that lower physical scores predict future frequent attendance at 12 and 24 months but low mental score is associated with frequent attendance at only 24 months but not at 12 months. Patients with low educational attainment (primary school or less) are more likely to be frequent attenders than others. This effect occurs at 12 and 24 months but not consistently. Although, secondary education is predictive of frequent attendance at 12 months, at 24 months it is only those who have vocational training and higher school or university education. Nevertheless, all those with greater than elementary school education are less likely to be frequent attenders. It is difficult to explain these findings and it is likely that these are chance findings or that our sample was too small to produce meaningful results. Our finding with respect to major depression, panic and anxiety disorder does not accord with the finding from a recent paper that suggested that somatoform disorders were significantly associated with frequent attendance behaviour after adjusting for depression, panic and anxiety disorders [16].

When assessing frequent attendance longitudinally regression towards the mean is likely to occur [17]. Nevertheless, the high rate of persistent frequent attendance is remarkable despite this phenomenon.

The main strengths of the study are its longitudinal design, and a random and representative sample of Slovenian family medicine practices’ attenders. The main limitation of our study is a considerable loss of participants to follow-up after 24 months (from 847 to 542 participants). The main reason for losses to follow up was that many family physicians did not want to participate in the review of the cohort in 2008. In Slovenia we still do not have universal electronic medical records which would ease access to data on frequent attendance. In 2005 there were more frequent attenders lost to follow up compared to the non-frequent attenders. In 2005 there were 30,7% of participants lost to follow up (233 participants of 760 participants in 2004). There were 37,2% of frequent attenders in 2004 lost to follow up in 2005 (35 frequent attenders of 94 frequent attenders in 2004).

Associations of lower educational level have already been reported in previous studies in Slovenian general practice [8], [9]. Further research is needed to explore the reasons for this. Our findings contradict the data from the literature [3], [5], [6], where frequent attendance is connected with psychiatric disorders and psychotropic drugs. We can only speculate the reasons for this. It is unlikely that people with mental disorders do not seek help for their problems in Slovenian family medicine practices and prevalence figures suggest that the rate of diagnosis is similar to that in other countries. Norton speculates that the lack of association with depression could be explained by the increased ability of family physicians to detect depression and therefore treat patients in primary care or refer them for specialist care [16].

A significant proportion of the workload of Slovenian family medicine physicians represents therapy and rehabilitation during the sick leave. There is a lot of administrative work required, even for uncomplicated illnesses. Even a self-limiting illness such as a common cold requires that an employed patient has to visit the practice at least twice to arrange sick leave. Perhaps this explains why employed patients were five times more likely to be classed as frequent attenders than the unemployed, students or housewives.

Slovenian general practitioners need to be aware of the workload involved in attenders with lower levels of physical well-being and the relative lack of frequent attendance that occurs in the case of people with psychological disorders. Larger studies should be done to confirm whether our research findings hold true and if so to explore the reasons for lack of an effect on attendance rates in people with psychological problems and the possible mechanism for frequent attendance in people with lower levels of education.

### Conclusion

We have found higher rates of persistent frequent attendance than described in the literature. There is strong and constant association of lower quality of life and lower educational level with future frequent attendance. It is still not clear if frequent attendance is a negative phenomenon or it is appropriate. If frequent attenders are sicker, could one not expect them to attend more frequently?
